# Cardiac CT Perfusion Imaging of Pericoronary Adipose Tissue (PCAT) Highlighting Potential Confounds in CTA Analysis

**DOI:** 10.3390/jcm14030769

**Published:** 2025-01-24

**Authors:** Hao Wu, Yingnan Song, Ammar Hoori, Juhwan Lee, Sadeer G. Al-Kindi, Wei-Ming Huang, Chun-Ho Yun, Chung-Lieh Hung, Sanjay Rajagopalan, David L. Wilson

**Affiliations:** 1 Department of Biomedical Engineering, Case Western Reserve University, Cleveland, OH 44106, USA; 2 Harrington Heart and Vascular Institute, University Hospitals Cleveland Medical Center, Cleveland, OH 44106, USA; 3 Department of Radiology, MacKay Memorial Hospital, Taipei 10449, Taiwan; 4 Division of Cardiology, Department of Internal Medicine, MacKay Memorial Hospital, Taipei 10449, Taiwan; 5 School of Medicine, Case Western Reserve University, Cleveland, OH 44106, USA; 6 Department of Radiology, Case Western Reserve University, Cleveland, OH 44106, USA

**Keywords:** cardiac CT perfusion, coronary CT angiography, pericoronary adipose tissue

## Abstract

**Background**: Features of pericoronary adipose tissue (PCAT) from coronary computed tomography angiography (CCTA) are associated with inflammation and cardiovascular risk. As PCAT is vascularly connected with coronary vasculature, the presence of iodine is a potential confounding factor on PCAT HU and textures that has not been adequately investigated. We aim to use dynamic cardiac CT perfusion (CCTP) to understand the perfusion of PCAT and determine its effects on PCAT assessment. **Methods**: From CCTP, we analyzed HU dynamics of territory-specific PCAT, the myocardium, and other adipose depots in patients with coronary artery disease. HU, blood flow, and radiomics were assessed over time. Changes from peak aorta time, P_a_, chosen to model the acquisition time of CCTA, were obtained. **Results**: HU in PCAT increased more than in other adipose depots. Blood flow in PCAT was ~23% of that in the contiguous myocardium. A two-second offset [before, after] P_a_ resulted in [4 ± 1.1 HU, 3 ± 1.5 HU] differences in PCAT, giving a 7 HU swing. Due to changes in HU, the apparent PCAT volume reduced by ~15% from the first scan (P_1_) to P_a_ using a conventional fat window. Comparing radiomic features over time, 78% of features changed >10% relative to P_1_. Distal and proximal to a significant stenosis, we found less enhancement and longer time-to-peak distally in PCAT. **Conclusions**: CCTP elucidates blood flow in PCAT and enables the analysis of PCAT features over time. PCAT assessments (HU, apparent volume, and radiomics) are sensitive to acquisition timing and obstructive stenosis, which may confound the interpretation of PCAT in CCTA images. Data normalization may be in order.

## 1. Introduction

Pericoronary adipose tissue (PCAT) assessment via coronary CT angiography (CCTA) has emerged as a novel CT imaging-based biomarker associated with plaque vulnerability, plaque-specific inflammation, and cardiovascular risk [1,2,3]. The fat attenuation index (FAI), related to the HU values, has been reported to be higher in lesions with higher stenosis severity and has been associated with vascular inflammation as proven from biopsy samples taken from patients undergoing cardiac surgery [4]. More recently, the prognostic value of FAI has been highlighted in predicting both major adverse cardiovascular events (MACEs) and rejection in heart transplant recipients [5]. The related FAI-score (Caristo Diagnostics) is a standardized metric for assessing FAI that accounts for “technical scan parameters (e.g., tube voltage), anatomical factors related with the fat distribution around the arteries and basic demographics (age, sex) [6]”. The FAI-score predicts cardiac mortality and MACE independently of other cardiovascular risk factors in patients with and without obstructive coronary artery disease (CAD) [7]. In additional reports, a high PCAT HU was found in lesions with higher stenosis severity, in lesions with higher rates of atherosclerosis progression, and in culprit vs. non-culprit lesions in patients presenting with acute coronary syndromes [1,8]. CCTA-based radiomics profiling of coronary artery PCAT detects perivascular structural remodeling associated with coronary artery disease and improves cardiovascular risk prediction [9,10].

These previous studies were all performed on CCTA images with iodine contrast, which could be a potential confounding factor in the assessment of PCAT. Although the blood flow in adipose is less than in other tissues, it is expected that the perfusion of iodine will elevate adipose HU values, with effects on other characteristics such as apparent volume and texture radiomics. Indeed, a recent study from Almeida et al. found that iodine contrast may affect the apparent volume of PCAT, and they demonstrated the feasibility of assessing PCAT from non-contrast images [11]. They found that before contrast injection, the PCAT HU value was lower, and the effective volume, as assessed using the traditional fat window (−190 HU, −30 HU), was increased. Continuing with this line of reasoning, we believe that there could be a dependence of HU, volume, and texture radiomics features in relation to the time since injection as the iodine contrast bolus moves through the vasculature and the presence of a flow-limiting stenosis. In addition, coronary artery motion, scan parameters (i.e., kVp), beam hardening effects from contrast agent, scanner type, scanner calibration, and body size may all potentially influence PCAT assessment.

In this report, we use dynamic CCTP to elucidate the role of iodine dynamics on PCAT assessment. We measure PCAT blood flow and determine changes in HU values of PCAT, in comparison to the myocardium, and other adipose depots. Using images at the time of peak contrast in the aorta (P_a_) to model a CCTA acquisition, we compared HU and radiomics assessment to determine the potential confound due to variable acquisition timing. As obstructed vessels will have reduced flows, we also compared PCAT with and without obstructive disease. Our goal is to elucidate the extent to which contrast enhancement with CCTA leads to uncertainty in conventional PCAT assessments.

## 2. Materials and Methods

### 2.1. Study Population

This study was approved as a retrospective study of de-identified data by the local institutional review board. Images were acquired starting in 2013 at Mackay Memorial Hospital, Taiwan, and shared under a data use agreement. We analyzed 9 patients with suspected coronary artery disease (CAD) who underwent CCTA and stress CCTP. We excluded patients based on the following criteria: (1) age < 20 years, (2) coronary artery bypass grafting, (3) acute or old myocardial infarction, (4) complete left bundle branch block, and (5) inadequate datasets such as poor image quality of CCTA and insufficient CCTP analysis. Sequential CCTA and stress dynamic CCTP were both performed using a dual-source CT system (Somatom Definition Flash; Siemens Healthineer, Forchheim, Germany) with a collimation of 64 × 2 × 0.6 mm and flying-focal spot, resulting in 2 × 128 sections.

### 2.2. CCTA Imaging Acquisition Protocol

CCTA acquisition and analyses are now described. A 20-gauge intravenous line was placed in the right antecubital vein for the administration of iodinated contrast medium and drugs related to stress perfusion. For CCTA, after a non-contrast localizing scout image was obtained, a timing bolus acquisition at the level of the aortic root was performed by administering a test bolus of 15 mL (flow rate, 5 mL/s) of contrast medium (Iopamidol 370; Bracco, Milan, Italy), followed by the administration of 20 mL of saline with a dual-syringe power injector (Ulrich medical CT motion, Ulm-Jungingen, Germany). The timing for the beginning of acquisition for coronary CTA was determined by adding 8 s to the time of peak contrast enhancement in the ascending aorta for adequate enhancing coronary arteries. Coronary CTA acquisition was performed with dual source, 120 kVp, and 320 mA per rotation as well as a 0.28 s gantry rotation time with 50 mL of contrast medium followed by 40 mL of saline injected using the power injector with an injection rate of 5.0 mL/s. Prospective ECG triggering was used to cover 70–80% of the R-R interval. Images were reconstructed with a medium smooth kernel (B26), a slice thickness of 0.5 mm, and an increment of 0.3 mm.

### 2.3. CCTP Imaging Acquisition Protocol

A stress cardiac CT perfusion was performed 10 min after finishing the CCTA scan. The total duration of the administration of dipyridamole (0.56 mg/kg) was 4 min. The stress dynamic CCTP was performed six minutes after the beginning of dipyridamole infusion. The dual-source scan was performed for 30 s, starting 6 s after the power injector started to administer the contrast medium. Scan parameters were dual source, 100 kVp, 320 mAs per rotation, and two alternating table positions were used in the prospective ECG-triggered mode, with the table moving forward and backward between the two positions (e.g., “shuttle mode”, with a table acceleration of 300 mm/s). Since the detector width was 38 mm with a 10% image overlap at the two positions, the imaging coverage was set to 73 mm. Images were acquired for every single heartbeat for the heart rate equal or less than 63 beats per minute and every second heartbeat for the heart rate of greater than 63 beats per minute. A total of 50 mL of contrast medium was injected using the power injector followed by 60 mL of saline, both at an injection rate of 5.0 mL/s. After stress CCTP, aminophylline was given (3 mg/kg) intravenously and delivered over 2 min.

### 2.4. Image Analysis

The CCTA data were evaluated by two experienced readers (CHY and WMH, with 16 and 6 years of cardiac CT experience, respectively). Readers were blinded to the subject’s clinical presentation and history. Any disagreement was solved through a consensus. On a per vessel basis, stenoses were classified as normal, 0% luminal diameter stenosis; minimal, 1% to 24% stenosis; mild, 25% to 49% stenosis; moderate, 50% to 69% stenosis; severe, 70% to 99% stenosis; and occluded, 100% stenosis. Stenoses > 70% were considered as obstructive stenosis.

**Figure 1:**
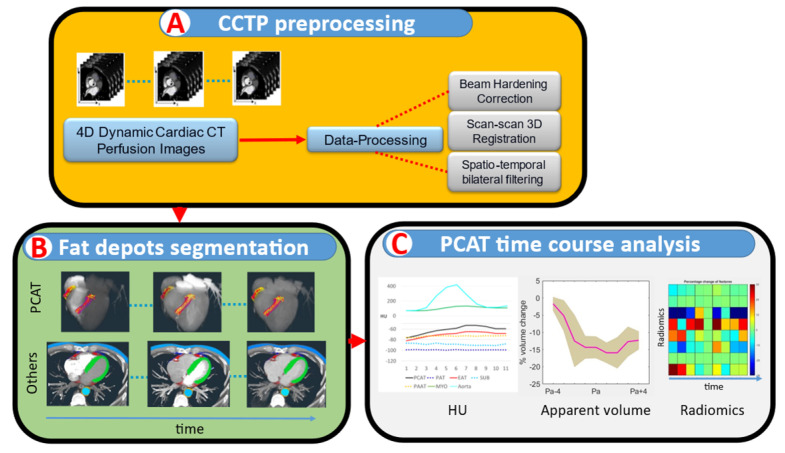
Pipeline of PCAT analysis in CCTP data. See text for description.

We used an in-house CCTP analysis software (Version 0.1) for CCTP image processing (Figure 1), as described in our previous publications [12,13,14]. Briefly, we performed a calibration-free beam-hardening correction for each scan [12]. To reduce the motion artifacts between scans, we performed a two-step registration (rigid body followed by non-rigid) to register all scans to the scan with peak enhancement of the aorta, as previously described [15]. We applied a spatio-temporal bilateral filter to the registered scans to further reduce noise.

### 2.5. Tissue Segmentation

The myocardium (MYO) and aorta were segmented automatically using U-Net [16]. ROIs for paracardial adipose tissue (PAT), epicardial adipose tissue (EAT), periaortic adipose tissue (PAAT), and subcutaneous adipose tissue (SUB) were manually segmented. To segment PCAT, the coronary lumen (LAD and RCA) was segmented manually. We segment PCAT around the LAD and RCA by using the aorta peak enhancement (P_a_) scan with prior visualization of coronary arteries. Two cardiology fellows specializing in cardiovascular imaging semi-automatically segmented and identified the centerlines of the two main coronary arteries (LAD and RCA) using an opensource software (3D slicer, version 5.6.1). Each image was initially processed by one resident, and the accuracy of the segmentation was subsequently verified by another person to ensure quality and consistency. We then created binary masks in axial images (deemed axial-disk-masks), which were centered on the centerline with a diameter 2 times the median effective diameter of the first 40 mm segmented lumen of coronaries. Finally, voxels in the axial-disk-mask within the fat window (−190 HU, −30 HU) were selected as PCAT. Blood flows were computed for the myocardium and PCAT region based on a super-voxel-based robust physiologic model [13,14].

PCAT features were extracted from voxels within the binary masks at each time point. We extracted both hand-crafted (13 features) and radiomics (8 features) from the PCAT region. For hand-crafted features, we focused on the histogram feature of PCAT HU values (e.g., skewness, kurtosis of the HU value histogram, and the probability in range of HU values) and the PCAT axial area features (e.g., mean area of PCAT in axial view). For the radiomics feature, we extract features that were sensitive in order to predict coronary artery disease reported by other studies via the PyRadiomics library [17,18,19]. We included shape features (e.g., original-shape-Flatness and original-shape-Elongation) and wavelet features (e.g., wavelet-LLH-firstorder-Mean and wavelet-LLL-glcm-Idmn). Texture features were calculated using PCAT voxels with 16-bin discretization. Radiomic features were extracted from both original PCAT images and three-dimensional wavelet transformations of the original image. Wavelet transformation decomposes the data into high- and low-frequency components, enabling the capture of discontinuities, ruptures, and singularities as well as the coarse structure of the data.

### 2.6. Statistical Methods

We statistically analyzed the features. ROIs include PCAT, subcutaneous adipose tissue (SUB), paracardial adipose tissue (PAT), epicardial adipose tissue (EAT), periaortic adipose tissue (PAAT), the myocardium (MYO), and the aorta. We examined temporal changes in attenuation and compared PCAT attenuation, apparent volume, and radiomics between time points. We accounted for variability of CCTP scan timing by using some key time points: (1). P_1_, the first time point which is the pre-contrast time point. (2). P_a_, the peak enhancement time point of the aorta, which is equivalent to the CCTA acquisition time point. (3). P_PCAT_, the peak enhancement time point of PCAT. These measurements are selected to minimize dependence on the exact timing of the CCTP acquisition, thereby reducing variability across the cohort. Each measurement was expressed as mean ± standard error. Paired Student’s *t*-test was used to compare the means between two groups. Differences were deemed statistically significant at *p* < 0.05. We used MATLAB statistic toolbox [20].

## 3. Results

**Figure 2:**
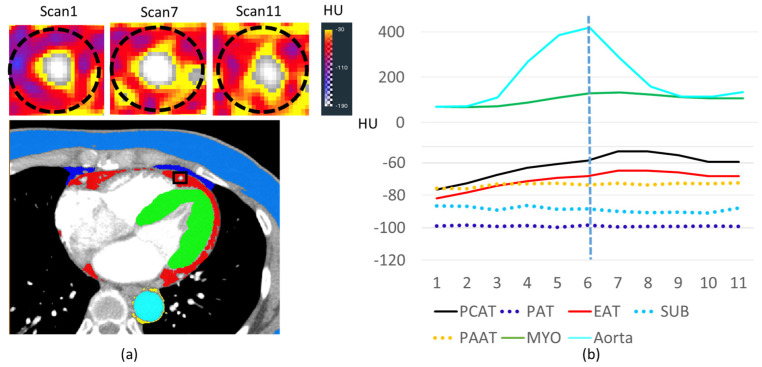
HU time courses in ROIs of a patient with unobstructed coronary arteries and adequate myocardial perfusion. Color-coded plots in (**b**) correspond to the ROIs identified in (**a**) **bottom**. In (**a**) **top**, HU values are shown in PCAT around the LAD at different scan times. HUs are elevated at scans 7 and 11 compared to scan 1. There are 11 scans comprising ~22 s in a shuttle mode acquisition. The vertical dashed line shows the scan of peak enhancement in the aorta, which we deem the CCTA imaging time point, P_a_. EAT = epicardial adipose tissue; HU = Hounsfield unit; MYO = myocardium; PAAT = periaortic adipose tissue; PAT = paracardial adipose tissue; PCAT = pericoronary adipose tissue; ROI = region of interest; SUB = subcutaneous adipose tissue.

**Figure 3:**
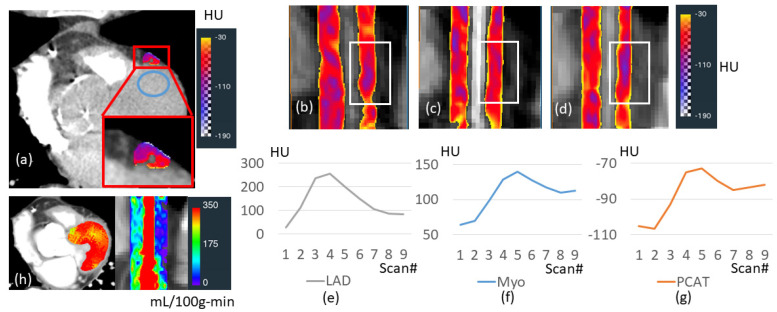
Comparison of PCAT enhancement to LAD and the nearby myocardium. CCTP image shows iodine in PCAT of LAD and myocardium (**a**). (See Supplementary Material Video S1 for dynamic iodine enhancement.) Curved-planar-reformatted images of LAD for scans 1, 5, and 9 are shown in (**b**), (**c**) and (**d**), respectively. Note the enhancement in the middle scan. Enhancement time courses of LAD, PCAT, and the nearby myocardium are remarkably similar in (**e**), (**f**), and (**g**), respectively. We computed the blood flow of myocardial and PCAT around LAD in (**h**) using our software described in Methods. Abbreviations are described in Figure 1.

Iodine enhancement was analyzed in various adipose depots for a patient with unobstructed coronary arteries and adequate myocardial perfusion (Figure 2). We found that adipose tissue, in general, showed much less enhancement than the myocardium. PCAT and EAT, however, enhanced much more than other fat depots and followed a time course like that of the myocardium. Other adipose tissues (PAT, SUB, and PAAT) had relatively flat attenuation curves. PCAT enhanced over time, giving a peak change of ~22 HU (P_1_: −75 ± 5 HU, P_PCAT_: −53 ± 6 HU, *p* < 0.05). We compared PCAT enhancement to that of ROIs in the LAD and the myocardium (Figure 3). The HU time course for PCAT was remarkably similar to that of the nearby myocardium. For this patient without obstructive coronary artery disease, the mean blood flow in PCAT was 23% of that in the myocardium (75 vs. 324 mL/100 g-min).

**Figure 4:**
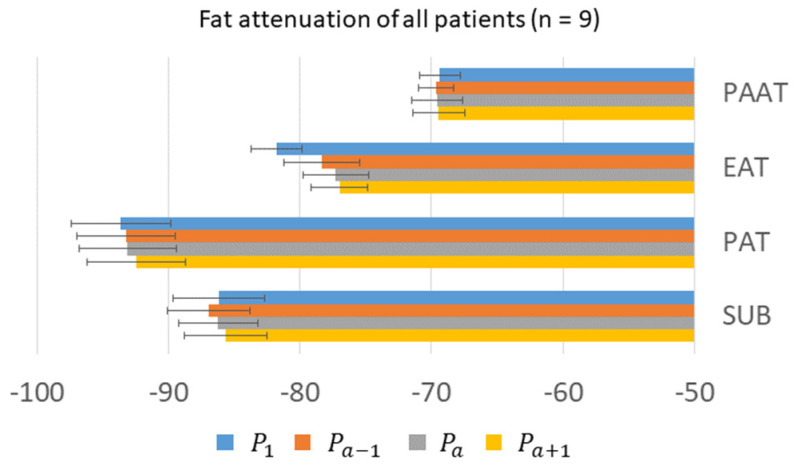
HUs of adipose ROIs at different times. HUs at the first scan (P_1_) vary greatly between ROIs, with the lowest value in pericardial adipose (PAT) and the highest in periaortic adipose (PAAT). Other times are relative to the time of peak enhancement of the aorta (P_a_). Epicardial adipose tissue (EAT) changes more than other ROIs. Abbreviations are the same as in Figure 1.

**Figure 5:**
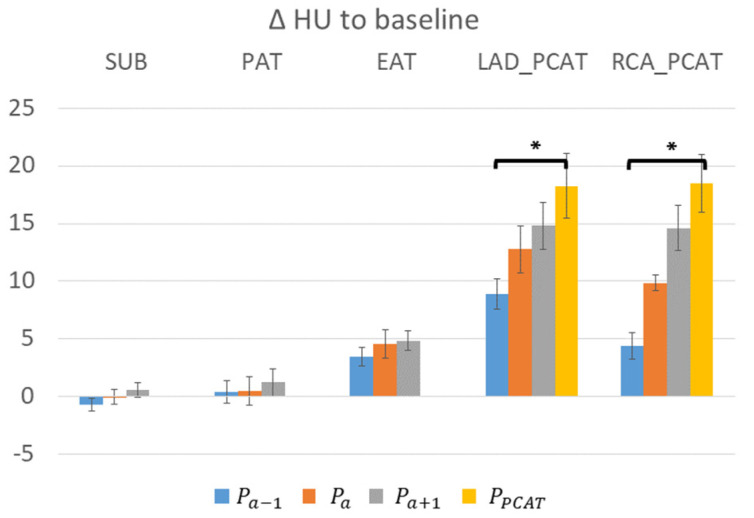
HU changes relative to baseline (P_1_) for various adipose depots and time points across 9 patients. ΔHU value order, PCAT > EAT > PAT ≥ SUB. PCAT is similar in LAD and RCA. Maximum enhancement (at P_PCAT_) is significantly different from P_a_, suggesting an enhancement delay in some hearts. Average time for P_PCAT_ is P_a+2_. Only vessels without obstructive disease are used (6 LADs and 6 RCAs). * *p* < 0.05. Abbreviations are described in Figure 1.

**Figure 6:**
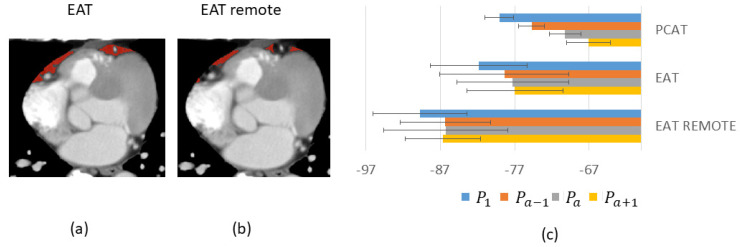
Regional HU values within EAT (containing PCAT and remote EAT). EAT and remote EAT ROIs are shown in (**a**) and (**b**), respectively. Baseline HU and changes with enhancement vary across ROIs, with PCAT changing the most (**c**). Only vessels without obstructive disease are used (6 LADs and 6 RCAs). Abbreviations are described in Figure 1.

We examined HU values in adipose tissue depots and determined the amount of variation that may be expected when sampling at different time points. In Figure 4, we showed that baseline HU values at P_1_ were different for different fat depots, with the highest value in PAAT, which was 24 ± 3 HU higher than that of PAT, indicating substantially different tissue characteristics. The change in HU in EAT was greater than in other types of adipose tissue. In Figure 5, across nine patients, we analyzed ΔHU values relative to P_1_, providing some correction for individual differences. Over all scans, ΔHU values were under 5 HU for fat depots SUB, PAT, and EAT. PCAT ΔHU values relative to P_1_, for both LAD and RCA, far exceeded those in other depots. In addition, changes relative to P_a_ may exceed 8 HU, underscoring that the timing of a CCTA acquisition may affect average HU values on PCAT. In addition, for PCAT around vessels without obstructive stenosis, the HU values were −74 ± 1.9, −70 ± 2.1, and −67 ± 2.9 at P_a−1_, P_a_, and P_a+1_, respectively, given a −4 ± 1.1 to 3 ± 1.5 HU difference with a two-second offset (before and after) in acquisition around P_a_, as shown in Figure 6c.

**Figure 7:**
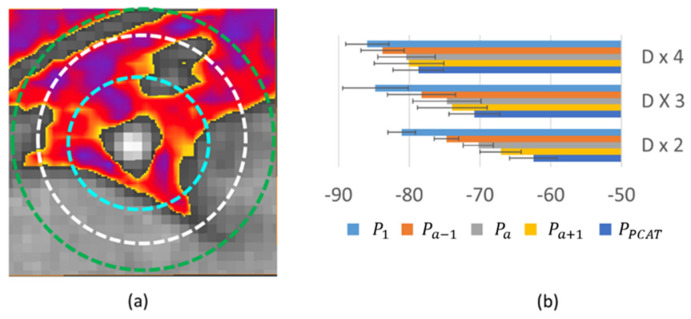
HU value of adipose as a function of radial distance from the vessel wall. The three non-overlapping ROIs are shown in (**a**). The diameter of blue, white, and green circles are 2 × D, 3 × D, and 4 × D, where D is the diameter of the vessel wall. HU values at different times for the different ROIs are shown in (**b**). Baseline HU is higher near the vessel wall. The enhancement change in HU increases near the vessel wall. Only vessels without obstructive disease are computed (6 LADs and 6 RCAs). Please note that the white circle (3 × D) roughly corresponds to the region of interest reported in the literature. Clearly, the 2 × D disk gives higher HU values than the 3 × D annular ring, indicating a spatial gradient from the vessel wall. In all regions, there is a dependence upon the frame since the time of injection. To determine if there was a time dependence of the spatial gradient, we analyzed the change (ΔHU) between average HUs in the 2 × D disk and the 3 × D annular ring as a function of the image frame. The ΔHU values were 3.7 ± 4.7, 3.6 ± 4.5, 4.4 ± 4.8, 6.8 ± 5.1, and 8.3 ± 5.1 HU for frames P_1_, P_a−1_, P_a_, P_a+1_, and P_PCAT_, respectively, again suggesting a dependence upon acquisition timing.

We analyzed EAT at varying distances from coronary arteries. In Figure 6, we analyzed “remote” EAT and compared results to all EAT and PCAT. Baseline values were over 9 HU higher for PCAT than remote EAT, suggesting substantially different characteristics (Figure 6c). In addition, PCAT demonstrated a greater change (~12 HU) with iodine enhancement, while remote EAT only changed about 3 HU, suggesting that PCAT is more substantively perfused. All EAT, including PCAT, shows a similar trend to PCAT but with a smaller change. In addition, we analyzed the enhancement of PCAT at different radial distances from the vessel wall to try to determine a potential rationale for the region of PCAT analysis having varying definitions, including “distance from the perimeter of the vessel equal to the diameter of the vessel [4]”. From Figure 7, we analyzed different disks and annular regions as defined in the figure. Baseline HU values were higher for the inner disk than for the two annular rings. In addition, ΔHU from P_1_ to P_PCAT_ progressively decreased as one goes out from the vessel wall.

**Figure 8:**
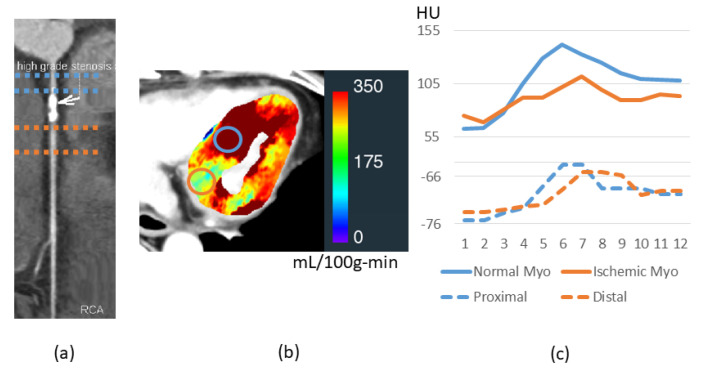
HU time courses in ROIs of a patient with an obstructive lesion and reduced myocardial blood flow in the RCA territory. Severe stenosis on the RCA (**a**) corresponds to reduced proximal myocardial blood flow. (**b**) Distal as compared to proximal PCAT ROIs [blue and brown regions in (**a**)] give a delayed and depressed enhancement time course (**c**-**bottom**), which is reasonably mirrored in the myocardial regions. Perfusion pressure proximal to the lesion will be increased relative to that found distally. Myo: myocardium.

Given that there is reduced myocardial blood flow in territories distal to significant obstructive stenosis, we wanted to determine if there were similar effects on PCAT (Figure 8). Distal to the lesion, there was a delay and a diminution of the peak, for both the myocardium and PCAT as compared to curves obtained proximal to the lesion. The diminution in peaks was present even when we evaluated ΔHU by subtracting baseline values. We found that four out of six vessels with severe stenosis followed a similar trend. Although there is no statistical significance, ΔHU between P_1_ and the peak enhancement for PCAT around proximal of a lesion is higher than those around the distal region of a lesion (16.1 ± 6.7 HU and 12.3 ± 3.4 HU, *p* = 0.2).

**Figure 9:**
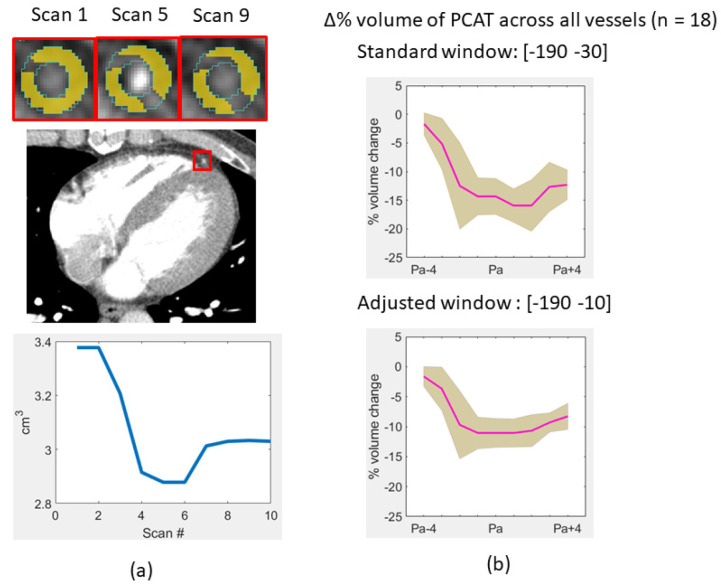
PCAT apparent volume changes over time. In (**a**), LAD PCAT apparent volume change (**bottom**) is shown for the patient (**above**) with non-obstructive disease and a fat threshold [−190 HU, −30 HU]. In (**b**), the percentage of apparent volume change relative to P_1_ is shown across all vessels with the conventional threshold and an adjusted threshold [−190 HU, −10 HU]. The solid line is the mean, and the shadow is the standard error. Percent apparent volume changes are substantial but reduced with the adjusted threshold.

In addition to the effect on HU, we investigated the role of iodine perfusion on other features of interest such as apparent PCAT volume (volume of voxels within the axial–radial disks falling within the standard fat window [−190 HU, −30 HU]). Using this definition, the apparent PCAT volume changed substantially over time (Figure 9). As voxels were “lost” due to their enhancement with iodine, we investigated an extended fat window [−190 HU, −10 HU]. In Figure 9a, the apparent PCAT volume at LAD for a patient changed from 3.4 cm^3^ to 2.9 cm^3^ from P_1_ to P_a_, giving a 14.7% reduction using the standard fat window. Using the extended fat window, the change was 12.3%. In Figure 9b, over all vessels (n = 18), the apparent PCAT volume at P_a_ changed by means of ~15% and ~11% relative to the volume at P_1_, for the standard and the extended fat windows, respectively.

**Figure 10:**
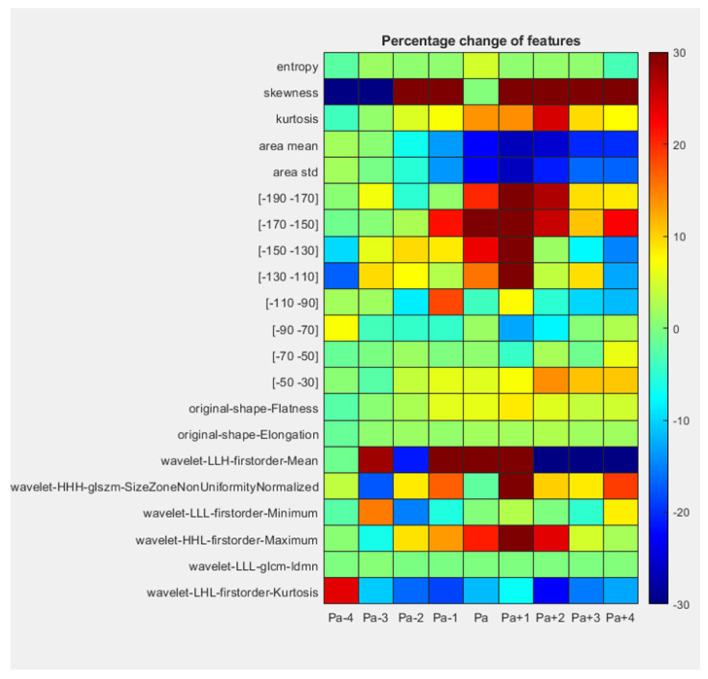
Changes in feature values with iodine perfusion. Twenty-one hand-crafted and radiomic library features are on the vertical axis. We present mean percent changes as compared to values at P_1_ from P_a−4_ to P_a+4_. The range of change was [−214%, 460%], but data were truncated to [−30%, 30%] for visualization. We analyzed vessel territories without an obstruction (n = 12).

In addition to volume, we considered that the presence of iodine could change other radiomic features, including texture, which would likely depend upon vessel filling. In Figure 10, we analyzed thirteen hand-crafted features and eight radiomic features as suggested in the literature [17,18] over time. Only five (22%) of the hand-crafted and radiomics features changed less than 10% relative to P_1_. They include entropy, original-shape-Elongation, original-shape-Flatness, wavelet-LHL-firstorder-Kurtosis, and wavelet-LLL-glcm-ldmn. Other features, such as area mean, change over time in a manner similar to volume, as described previously. Note that a change of ±2 scan intervals from P_a_ can dramatically change feature values, suggesting a dependence on the CCTA acquisition time, e.g., see wavelet-HHH-glszm-SizeZoneNonUniformityNormalized.

## 4. Discussion

In this work, we have provided important new information on the influence of iodine contrast on PCAT assessment. The use of cardiac CT perfusion (CCTP) provides substantial clarity on the influence of contrast kinetics on PCAT assessment in a number of ways. First, PCAT substantively enhances contrast with iodine in concert with the myocardium, with a blood flow of about one-fifth of that of the myocardium. Other adipose depots (e.g., paracardial, subcutaneous, and periaortic) show little, if any, enhancement. (This could be due to both reduced vascularization as compared to PCAT, vascular connections to the myocardium, and spreading of the iodine bolus in feeding arteries of other adipose tissues.) Together, these observations suggest that unlike other depots, PCAT and EAT are in vascular communication with the myocardial and coronary tissues. Second, dynamic PCAT enhancement with iodine confounds HU, apparent volume, and radiomic features, depending on the timing of the CCTA acquisition. Third, the interpretation of PCAT assessment may be further influenced by the presence of obstructive coronary disease.

Our results also provide new information on the vascularity of PCAT, when contrasted with other adipose depots. For instance, we found that the HU time course in EAT demonstrated considerably more enhancement than other types of adipose tissue outside the pericardium (Figure 2, Figure 4 and Figure 5). This finding is consistent with results in the literature, suggesting that blood flow in visceral adipose tissue far exceeds that of subcutaneous adipose tissue [21]; this is perhaps related to the underlying metabolic requirements. The EAT would be expected to demonstrate higher perfusion, given the underlying metabolic requirements of the heart contiguous to it with a shared vascular supply. Imaging of the vasa vasorum shows dense vascularization [22], much of which would be in contact with the surrounding tissues, including adipose [21], supporting our observation of increased enhancement of PCAT near the coronaries that drops rapidly within a distance of two-vessel diameters (Figure 7). Given the metabolic requirements of the coronary arteries (which in turn feed the myocardium), the importance of subserving flow to the coronary artery wall and surrounding adipose cannot be overstated. PCAT has been noted to have a different transcriptomic signature than that of other EAT regions [23].

The timing of a CCTA acquisition can also affect multiple assessments including HU values, apparent volume, and radiomics which has important implications for studies that report these characteristics without consideration of contrast kinetics. A two-second offset [before, and after] in acquisition around P_a_ results in differences [−4 ± 1.1 to 3 ± 1.5 HU difference] in PCAT attenuation. We observed up to a ~15% apparent volume difference between the pre-contrast scan and the peak time point with the standard fat window [−190 HU, −30 HU]. A reduced change in apparent volume (~11%) was observed when using the adjusted window [−190 HU, −10 HU], indicating that some PCAT voxels were enhanced by iodine and exceeded −30 HU. These findings underscore the importance of accounting for temporal dynamics of iodine enhancement, which previous studies did not address. For example, studies have reported variable PCAT HU values associated with CAD. Lin et al. reported that PCAT HU around the RCA was approximately −91 and −96 HU for stable CAD and a control group, respectively [8], whereas Wen et al. found that PCAT HU was approximately −66 and −75 HU for vessels with FFR < 0.8 and FFR > 0.8, respectively [24]. These discrepancies might be partly affected by variations in contrast timing, as demonstrated by our findings, which show substantial effects of iodine dynamics on HU, apparent volume, and radiomic features. By analyzing 21 hand-crafted and radiomic features in PCAT at different time points, we found that only 22% of hand-crafted and radiomics features changed less than 10% relative to the pre-contrast scan, emphasizing the need for standardized acquisition protocols to enhance consistency and comparability in PCAT assessments. Given the success of the FAI-score described previously, it would be interesting to know if it is immune to iodine bolus dynamics.

The presence of a proximal obstructive coronary artery stenosis further impacts PCAT HUs. In general, we found that HU values of PCAT were lower, and the time to peak enhancement was longer in PCAT regions distal to the stenosis, consistent with a reduced flow in the region and a reduced perfusion of the distal subtended myocardium. In earlier reports, PCAT HU was higher in the presence of CAD, defined as >50% stenosis, with increases of ~3 HU [4] and ~5 HU [24]. These studies did not interpret these values with regard to contrast timing. In more recent reports, authors have examined more severely obstructed vessels. Ma et al. reported that the lesion-specific PCAT mean HU value was ~4 HU lower in vessels with >70% stenosis than in vessels with <25% stenosis [25]. They argued that there might be more stable calcification and less inflammation in the presence of a severe stenosis as compared to mild and moderate stenoses. While these conjectured scenarios seem attractive from a biologic perspective, it is hard to support them without rigorous adjustments and consideration of other plausible hemodynamic considerations. For instance, the pressure drop across an obstructive stenosis may reduce flow to the distal region, effectively reducing microcirculatory flow to the coronary microvessels and coronary vasa vasorum distal to the stenosis. This is indeed evidenced by our results which demonstrate a reduction in distal myocardial perfusion and reduced enhancement in the distal PCAT. In the aforementioned CCTA studies, it is unclear to what extent their observed differences relate to hemodynamic considerations.

In addition to identifying potential confounding factors, our analysis with CCTP might lead to improvements in the assessment of PCAT. As vessel density (vascularity) and vessel leakiness (reflective of neo-angiogenesis) might change with inflammation [26], perfusion metrics (e.g., blood flow and retention) might be important features for risk prediction and may indeed capture some of these features. In addition, the additional analysis of CCTP data might lead to ways to normalize responses (e.g., normalizing on the aorta or vessel HU values) for improved characterization.

We admit several limitations including the single-center nature of this study with a predominantly Asian patient population. However, there is no reason to believe that these aspects would detract from the broader relevance of our findings. There could be residual iodine from the CCTA study during CCTP, but the 10 min delay between studies should marginalize any such effect [27]. Furthermore, ΔHU values during CCTP should not be affected by the presence of any baseline iodine. Our CCTP acquisitions, which differ from CCTA imaging with regard to kVp, iodine bolus size, acquisition method (e.g., shuttle versus spiral), and reconstruction are issues worth considering. To confirm the robustness and generalizability of our findings, we recommend future validation in larger, multi-center cohorts encompassing more diverse populations and imaging protocols. Such studies would help ensure that the effects of acquisition timing and PCAT perfusion metrics observed in this study hold across different clinical settings and patient demographics. Such studies would only be performed when CCTP is applied for the determination of ischemia. Since our CCTP methodology elucidates the influence of a number of factors critical to the interpretation of PCAT attenuation and radiomics, our study findings have direct relevance to the field.

## 5. Conclusions

Our findings provide new insights into PCAT perfusion and its assessment in CCTA images, emphasizing the need to account for acquisition timing and the presence of significant obstructive coronary stenosis. These factors can significantly influence PCAT attenuation (HU values), apparent volume, and radiomic features, potentially confounding their interpretation as biomarkers for coronary artery disease. By highlighting the impact of iodine dynamics and acquisition protocols, our study underscores the importance of standardizing imaging practices to improve the consistency and reliability of PCAT assessments. These findings have important clinical implications, as they can inform the optimization of diagnostic workflows, guide additional investigations in patients, and refine risk stratification models.

## Data Availability

Data available upon request due to privacy restrictions.

## Supplementary Materials

The following supporting information can be downloaded at the following website: https://www.mdpi.com/article/10.3390/jcm14030769/s1, Video S1: Dynamic iodine enhancement in the PCAT region.
